# Tideglusib promotes wound healing in aged skin by activating PI3K/Akt pathway

**DOI:** 10.1186/s13287-022-02949-2

**Published:** 2022-06-21

**Authors:** Jiachen Sun, Hongqing Zhao, Chuan’an Shen, Shiyi Li, Wen Zhang, Jinglong Ma, Zhisheng Li, Ming Zhang, Jianqiu Yang

**Affiliations:** Department of Burns and Plastic Surgery, The Fourth Medical Center of Chinese PLA General Hospital, Beijing, 100048 China

**Keywords:** Aging, Epidermal growth factor receptor (EGFR), Tideglusib, Apoptosis, Proliferation, Wound healing

## Abstract

**Background:**

Aging disturbs the skin morphology and function, manifested as thinned epithelium and impaired wound healing. As a major type of skin cells, epidermal stem cells (EpiSCs) are inevitably affected by aging. The effect of age on EpiSCs and wound healing needs to be further explored.

**Methods:**

Skin RNA-seq data of young (5 months) and old (30 months) CB6F1 mice were obtained from GEO Series GSE35322 with 10 in each age group. Differentially expressed genes were analyzed, and EpiSCs-related pathways were enriched by KEGG. The age-related changes of the screened PI3K/Akt pathway were validated by Western Blot and immunofluorescence of epidermis of SD rats (2, 17, and 23 months, *n* = 6). The expression of upstream protein EGFR was assessed by immunofluorescence in skin of mice (4, 13, and 23 months, *n* = 6) and human (respectively, 23, 28, 30 years old in the young group and 69, 73, 78 years old in the old group) skin. Inhibitors of EGFR were used to verify its effects on EpiSCs and wound healing. The small molecule drug Tideglusib was tested for its effects on signaling pathways of EpiSCs and wound healing of aged rats. Western Blot was used for the detection of signaling pathways in in vitro experiments. Cell migration assays were used to assess cell migration ability. Flow cytometry was used to detect changes in cell cycle and apoptosis levels. Sulforhodamine B assay and CCK-8 assay were used to evaluate cell proliferation and viability, respectively. Student’s *t* test and one-way analysis of variance (ANOVA) followed by the multiple comparisons Bonferroni test were used for statistical analysis. The 0.05 level of confidence was accepted as a significant difference.

**Results:**

EpiSCs-related PI3K/Akt pathway was enriched by KEGG and verified by decreased phosphorylation of Akt (32.1 ± 13.8%*, P* < 0.01) and mTOR (38.9 ± 11.8%, *P* < 0.01) in aged epidermis of rats. Furthermore, the expression of PI3K/Akt-upstream EGFR decreased with age in the epidermis of mouse (27.6 ± 5.5%, *P* < 0.01) and human (25.8 ± 9.3%, *P* < 0.01). With EGFR blocked by Erlotinib, EpiSCs showed reduced phosphorylation of Akt (30.4 ± 10.6%, *P* < 0.01) and mTOR (39.8 ± 12.8%, *P* < 0.01), impaired proliferation and migration after incubated for 24 h and 36 h (*P* < 0.05), and higher levels of apoptosis (11.9 ± 1.7%, *P* < 0.05), and rats showed slower wound healing from d7 to d14 after wounding (*P* < 0.01). In addition to slower wound healing rates, aged rats also showed a decrease in the efficacy of EGF, partly due to the downregulated EGFR expression. By activating PI3K/Akt pathway, Tideglusib promoted the proliferation and migration of EpiSCs with apoptosis inhibited (*P* < 0.01) and accelerated wound healing in aged rats from d7 to d14 after wounding (*P* < 0.05). Notably, the combined use of Tideglusib and EGF could further enhance wound healing in aged rats.

**Conclusions:**

The decreased expression of EGFR in epidermis with age resulted in decreased activity of the PI3K/Akt pathway and limited EGF efficacy. Tideglusib could assist wound healing in aged rats via activating PI3K/Akt pathway, which may be considered as an ingredient for medical and cosmetics use.

## Introduction

As the key physical barrier to the external environment, the epidermis prevents loss of water, solutes, and other components of the body and protects the body from potentially hazardous threats. To maintain proper tissue homeostasis and barrier function, the epidermis is constantly renewed and repaired by epidermal stem cells (EpiSCs) that proliferate, differentiate, and migrate to the stratum corneum [1].

After injury, a wound healing response is rapidly triggered to repair the epidermis and restore the skin barrier. Wound healing is a complex biological process which involves both resident and migratory cell populations, extracellular matrix, and the action of soluble mediators [2]. During re-epithelialization, the damaged skin is exposed to pathogens, and delays in the process can lead to higher incidence of infection and thus chronic wound formation.

Inevitably, the ability of tissues to repair and reconstruct declines with age [3–6]. Skin is particularly vulnerable to senescence, manifested as increased dryness, roughness, delayed wound healing, and increased susceptibility to infection. Studies have revealed that poor wound healing in aged adults is rooted partly in the reduced proliferative and migratory behavior of EpiSCs, which may be attributed to the disturbed gene transcription and protein expression [7, 8].

At present, in addition to routine disinfection and dressing change, the clinical treatment of wounds also includes the use of growth factor drugs such as EGF, which could promote the proliferation and migration of EpiSCs. Wounds of the young usually heal well under such treatments, while those of the old usually heal much more slowly, even with EGF. In view of the fact that the mechanism of skin aging has not been fully elucidated, there are currently no therapeutic drugs targeting wound healing in the elderly in clinical practice. Some studies have focused on promoting the healing of aged wounds through mesenchymal stem cells (MSCs) and their exosomes. However, considering the difficulty of obtaining sufficient number of highly active autologous MSCs in the elderly, the immune rejection of allogeneic MSCs, or the complex components of exosomes, it is very meaningful to find a small molecule targeted drug that can promote wound healing in the elderly [9].

In the present study, we were drawn to analysis skin transcriptome sequencing data of different ages to reveal targets that may be associated with the delayed wound healing in the elderly. In view of the important role of EGF in EpiSCs and wound healing, the age-related changes of EGF-related signaling pathway and its receptor protein EGFR were emphatically analyzed in vivo. Further, EGFR blockers were used to explore its effect in EpiSCs and wound healing of young rats. In order to examine the age-related efficacy changes of EGF, wound experiments in rats of different ages were carried out. Meanwhile, the small molecule drug Tideglusib, a membrane receptor-independent activator of the PI3K/Akt pathway, was also applied to EpiSCs and wounds of different ages to testify its efficacy especially on aged wounds.

## Materials and methods

### Study design

The experiments were conducted in four parts.

Section 1: Based on the age-related PI3K/Akt pathway found by the analysis of sequencing data, epidermis samples of rats of different ages were exposed to Western Blot for detection of the PI3K/Akt pathway. After confirming the age-related decline in PI3K/Akt pathway activity, the expression of EGFR, the upstream of the PI3K/Akt pathway, was further detected in rat, mouse, and human skin samples of different ages. With the age-related expression change of EGFR confirmed, we conducted wound experiments in young rats with the inhibitor of EGFR to confirm the relationship between EGFR function and wound healing.

Section 2: Considering that EGFR is a membrane receptor necessary for EGF to function, so we conducted wound experiments in rats of different ages to explore whether the reduced EGFR expression in the skin of aged rats would affect the efficacy of EGF.

Section 3: After confirming the effect of Tideglusib on PI3K/Akt pathway of EpiSCs in in vitro experiments, we took rats of different ages to conduct wound experiments to observe the therapeutic effect of Tideglusib on rats of different ages and the activity of PI3K/Akt pathway during wound healing.

Section 4: Considering the limited effect of EGF in wound repair in aged rats, and Tideglusib could activate the PI3K/Akt pathway independent of membrane receptors, we took EpiSCs and aged rats to observe whether the combined use of these two drugs could promote wound healing better.

### RNA-seq data analysis

Skin RNA-Seq data of young (5 months) and old (30 months) CB6F1 mice were obtained from GEO Series GSE35322 with 10 in each age group (https://www.ncbi.nlm.nih.gov/geo/query/acc.cgi?acc=GSE35322). Analysis of RNA-seq data was done using the DESeq2 package in R with a screening criterion of *P*.adj < 0.05. Upregulated or downregulated differentially transcripts were enriched via Kyoto Encyclopedia of Genes and Genomes (KEGG) to find functional KEGG annotations.

### Rats and wounding experiments

Male SD rats (2-month-old, 17-month-old, and 23-month-old) were purchased from the SPF (Beijing) Biotechnology Co., Ltd., and raised in the Laboratory Animal Research Center (LARC) in accordance with institutional guidelines for one week before the experiment. All rats were maintained separately in a specific pathogen-free (SPF) animal room at a constant temperature (23 °C) and humidity (60%) with a 12-h light/dark cycle. We specified rats with “good hair coats” to avoid animals with clear signs of dermatitis, fighting, scratching, and inflammation. The tumor-free rats were screened for experiments. Totally, 75 2M rats, 45 17M rats, and 105 23M rats were used in the experiments.

For wounding experiments, dorsal hair was cut with clippers and skin was swabbed with EtOH prior to wounding. After animals were anesthetized with isoflurane using a gas anesthesia machine, depilation was performed and 10-mm biopsy punches were used to make two full-thickness (epidermis + dermis) wounds on both sides of the midline of the back as described, and 12-mm-diameter blue fenestrated sheets were used as a reference [7]. PBS was used in control groups. Intraperitoneal injection of Erlotinib (50 mg/kg, HY-50896, MCE) daily was used in 2M rats to evaluate the effect of blocked EGFR on wound healing. 10 μg/mL EGF (P00033, Solarbio) or 20 μM Tideglusib (HY-14872, MCE) was configured in PBS with a final volume of 200 μL and sprayed evenly on the wound of 2M, 17M, and 23M animals daily. Scab was removed for better wound area estimation and drug absorption. With 15 rats in each treatment group, 3 rats with 6 wounds were selected by random number table method for recording wound healing status by taking photographs at indicated time point. The wound healing process was quantified by using digital images evaluated with ImageJ software. The wound healing rate was calculated as (1 − residual wound area/total wound area) × 100%. In addition, 3 randomly selected rats were killed, respectively, at d0.5, d3, d7, d10, and d14 after wounding with 6 wound margin tissues collected for the detection of signal pathway. Procedures were performed using IACUC-approved protocols that adhere to NIH standards.

### Western Blot

**Fig. 1:**
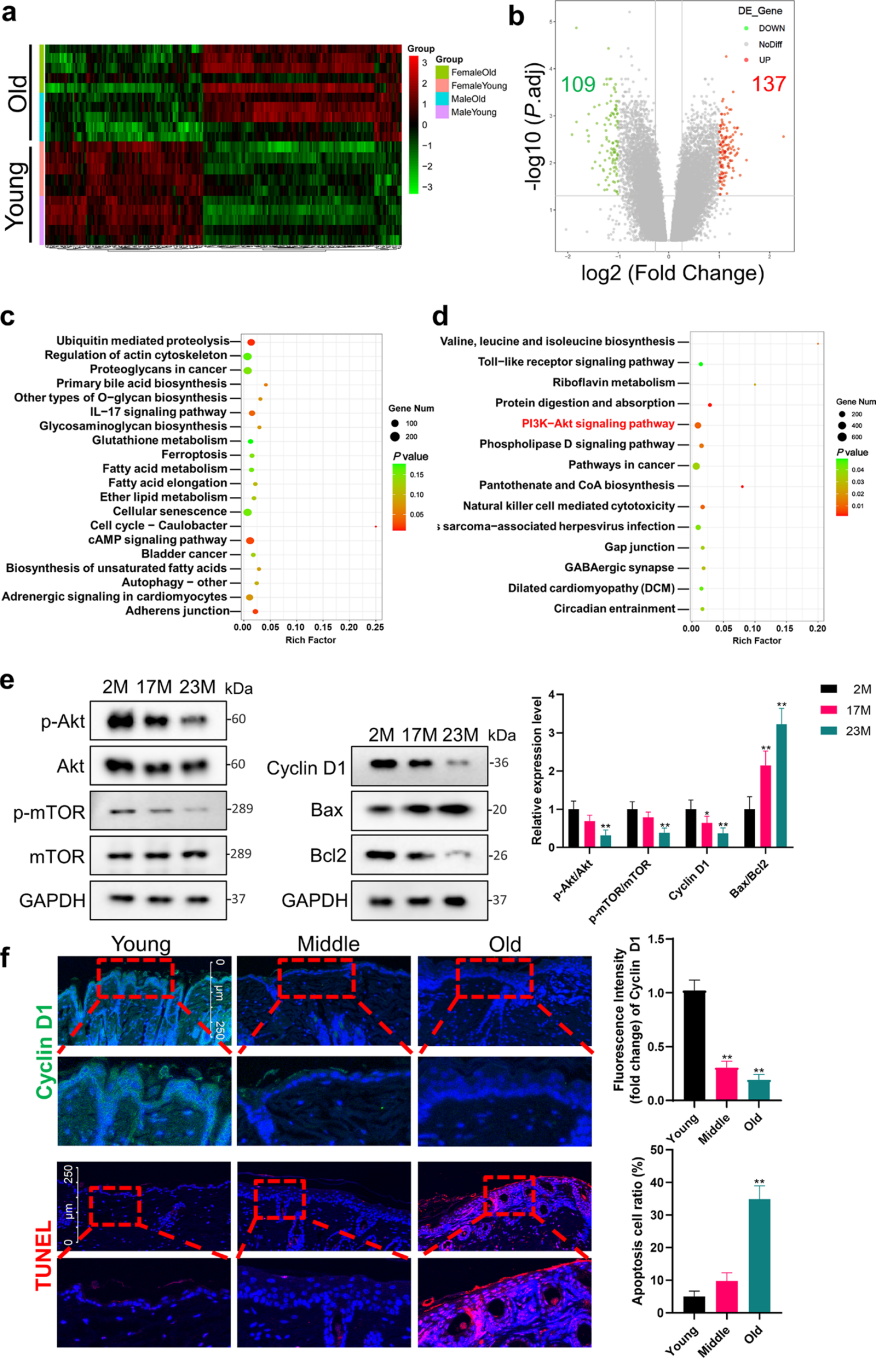
Transcriptome changes in skin aging and age-related changes in Akt/mTOR pathway. **a**–**d** The skin RNA-seq data of young (5 months) and old (30 months) CB6F1 mice (from GEO series GSE35322) were analyzed with the screening criteria: *P*.adj < 0.05. Number of animals: 10 for each age group, containing 5 males and 5 females. **a** Heat map of differentially regulated transcripts between groups. Green color denotes low FPKM expression, red high FPKM expression. **b** Volcano plot of differentially regulated genes between groups. Dots with fold changes greater ± two fold and *P*.adj < 0.05 were marked red or green. **c**, **d** The upregulated and downregulated genes were enriched by KEGG and displayed in the scatter diagram. **e** Western Blot was used to measure the phosphorylation of Akt and mTOR in epidermis from the back of 2 M, 17 M and 23 M rats. Proliferation ability is compared by Cyclin D1. The apoptosis level is indicated by the ratio of Bax to Bcl2. *N* = 6. *P < 0.05, **P < 0.01 versus the 2 M group. **f** Immunofluorescence images of young, middle and aged mice skin labeled with antibodies against Cyclin D1 [secondary antibodies are color-coded as shown]. TUNEL staining is used to mark apoptotic cells. Sections were co-stained with DAPI (blue) to visualize nuclei. *N* = 6. Scale bar, 250 μm. The box area besides each photomicrograph shows an enlarged version of the red square. *P < 0.05, **P < 0.01 versus the young group

**Fig. 2:**
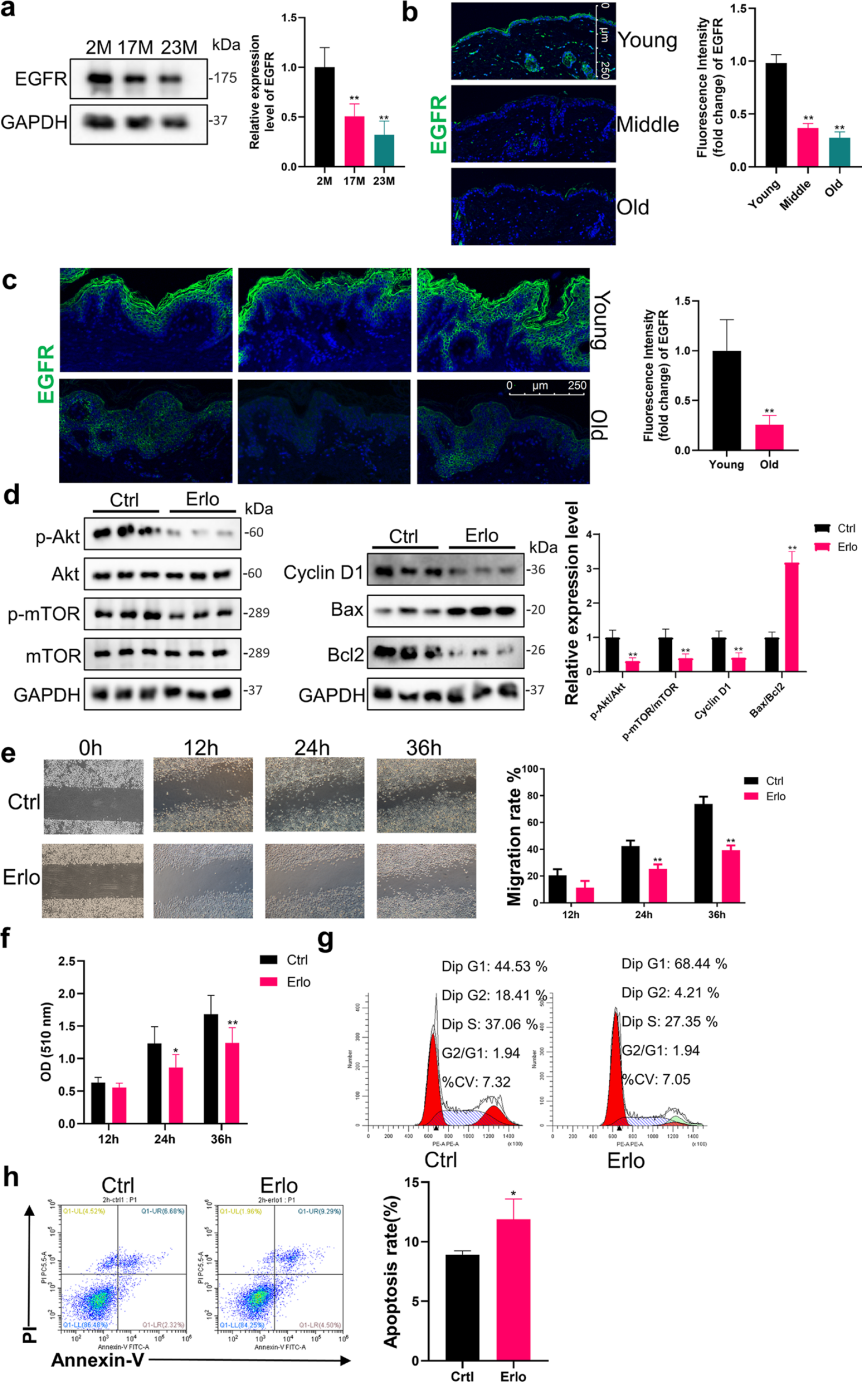
Declines in EGFR expression restricted the activation of PI3K/Akt in EpiSCs. **a** Western Blot was used to measure the expression level of EGFR in epidermis from the back of 2 M, 17 M and 23 M rats. *N* = 6. *P < 0.05, **P < 0.01 versus the 2 M group. **b** Immunofluorescence images of young, middle and aged mice skin labeled with antibodies against EGFR [secondary antibodies are color-coded as shown]. Sections were co-stained with DAPI (blue) to visualize nuclei. *N* = 6. Scale bar, 250 μm. *P < 0.05, **P < 0.01 versus the young group. **c** Immunofluorescence images of young and old human skin labeled with antibodies against EGFR [secondary antibodies are color-coded as shown]. Sections were co-stained with DAPI (blue) to visualize nuclei. *N* = 3. Scale bar, 250 μm. *P < 0.05, **P < 0.01 vs. the young group. **d** EpiSCs were randomly divided into two groups that incubated with PBS or 2 μM Erlotinib dissolved in Epilife medium for 24 h. Western Blot was performed. *N* = 3 for each group. *P < 0.05, **P < 0.01 vs. the control group. **e** Confluent EpiSCs were randomly divided into two groups that received PBS or 2 μM Erlotinib dissolved in Epilife medium and subjected to in vitro scratch-wound assays. *N* = 3 for each group. *P < 0.05, **P < 0.01 versus the control group at time-point. **f** SRB assay was used to evaluate the proliferation ability of EpiSCs treated with PBS or 2 μM Erlotinib. *N* = 6 for each group. *P < 0.05, **P < 0.01 vs. the control group at time-point. **g** and **h** Flow cytometry was further used to quantify cell cycle distribution and apoptosis in cells treated with PBS or 2 μM Erlotinib for 24 h. *P < 0.05, **P < 0.01 vs. the control group. *N* = 3 for each group

For tissue samples, skin samples and wound tissues were isolated from the backskin of rats with six samples in each group. Subcutaneous fat was removed from skin samples with a scalpel, and skins were placed dermis side down in 2.4 U/mL Dispase II (17105041, Gibco) for 1 h. Then the skins were scraped to separate the epidermis from the dermis. Epidermis was immediately frozen in liquid nitrogen. Frozen tissues were homogenized using tissue grinder and collected in RIPA Lysis and Extraction Buffer (89901, Thermo Fisher) with protease inhibitors (HY-B0496, MCE) and phosphatase inhibitors (HY-K0021, HY-K0022, MCE). For cell samples, the treated cells were washed with pre-chilled PBS and then collected in lysis buffer. After 10 min lysing on ice, tissue and cell debris were removed by centrifugation. Protein concentrations were measured using a bicinchoninic acid (BCA) protein assay kit (PC0020, Solarbio). The lysate was boiled for 5 min in 5 × Sodium dodecyl sulfate (SDS) loading buffer (P1040, Solarbio) containing 5% β-mercaptoethanol (444203, Millipore). Sixty micrograms of samples were then subjected to SDS–polyacrylamide gel electrophoresis (PAGE) on 10% Hepes-Tris Precast-Gel (PG01010-S, Solarbio) and transferred to polyvinylidene difluoride (PVDF) membranes (IPVH00010, Millipore). After blocked with 5% non-fat milk in Tris-buffered saline Tween (TBST) (pH 7.6) for 1 h, the membranes were incubated with corresponding primary antibodies overnight at 4 °C. Then, membranes were incubated with the appropriate horseradish peroxidase (HRP)-conjugated secondary antibodies at room temperature (RT) for 2 h. The antibody incubations were then followed by three times 5-min TBST washes. The protein bands were detected with ChemiDoc XRS chemiluminescence imaging system (Bio-Rad), quantified by Image Lab, and normalized to GAPDH levels or α-Tubulin. The experiment was performed 6 times for epidermis tissues or 3 times for cell samples. Relative expression levels were calculated based on the expression level of the 2M groups or the control groups. In Figs. 1e, 2a, d, and 4b, the expression of each group was compared with the 2M (control) group, respectively. In Figs. 2b, 5b, and 7c, the expression of each group was compared with the control group, respectively, at each time point. All antibodies used were as follows: GAPDH Rabbit Ab (5174, 1:1000, CST), α-Tubulin Rabbit Ab (2125, 1:1000, CST), Akt Rabbit Ab (4685, 1:1000, CST), Akt (phospho Ser473) Rabbit Ab (4060, 1:1000, CST), mTOR Rabbit Ab (2983, 1:1000, CST), mTOR (phospho S2448) Rabbit Ab (5536, 1:1000, CST), Cyclin D1 Rabbit Ab (ab16663, 1:200, Abcam), Bax Rabbit Ab (14796, 1:1000, CST), Bcl-2 Rabbit Ab (3498, 1:1000, CST), EGFR Rabbit Ab (ab52894, 1:200, Abcam), HRP-conjugated Goat Anti-Rabbit IgG(H + L) (SA00001-2, 1:1000, Proteintech).

### Immunofluorescence

Male C57BL/6 mice (4, 13, and 23 months) with 6 animals in each age group were purchased from the SPF (Beijing) Biotechnology Co., Ltd., and raised in the LARC in accordance with institutional guidelines. Backskin samples of C57BL/6 mice of different ages were collected after animals were anesthetized with isoflurane. Six human skin samples were obtained from patients who had undergone plastic and burns surgery in Fourth Medical Center of Chinese PLA General Hospital. With informed consent, about 3 × 3 mm skin specimens were collected , respectively, from 23-, 28-, and 30-year-old men as the young group and 69-, 73-, and 78-year-old men as the old group. Skin samples from mice and human were fixed in 4% paraformaldehyde (P0099, Beyotime). After embedded by paraffin, tissues were cut into slices, antigen repaired with proteinase K (P1120, Solarbio), permeabilized 10 min with 0.1% Triton X-100 (HFH10, Invitrogen) in PBS, and then blocked for 1 h in blocking buffer (2.5% normal goat serum, 0.3% Triton X-100). Primary antibodies (and their dilutions) used were as follows: EGFR Rabbit Ab (ab52894, 1:200, Abcam), Cyclin D1 Rabbit Ab (ab16663, 1:200, Abcam). Primary antibodies were diluted in blocking buffer and incubated at 4 °C overnight. After washing with PBS, secondary antibodies conjugated with Alexa 488 (ab150073, 1:200, Abcam) were added for 1–2 h at RT. Slides were washed with PBS and stained with Antifade Mountant with 4',6-diamidino-2-phenylindole (DAPI) (P36962, Invitrogen). Images were acquired with a confocal microscope (SP8, Leica). The mean fluorescence intensity in six randomly chosen areas was calculated by ImageJ. The mean fluorescence intensity of DAPI was used as a reference. Relative expression levels were calculated based on the expression level of the young groups. And each group was compared with the young group, respectively.

### TUNEL staining

Briefly, the in situ cell death detection kit (C1089, Beyotime) was used to measure apoptosis in the dorsal skin samples of mice (4, 13, and 23 months) with 6 animals in each age group according to the manufacturer's instructions. Skin samples from mice were fixed in 4% paraformaldehyde (P0099, Beyotime) for 24 h, then embedded by paraffin, and cut into slices. The slices were treated with 20 μg/mL proteinase K (P1120, Solarbio) at 37 °C for 30 min for antigen retrieval and washed with PBS for 3 times. Then, the slices were dyed using the TUNEL reaction solution prepared in a humid dark box for 1 h at 37 °C. After washing with PBS, the tissues were dyed using Antifade Mountant with DAPI (P36962, Invitrogen). Images were acquired with a confocal microscope (SP8, Leica). The apoptotic index was counted and calculated as an average of six samples by the ratio of TUNEL-positive cells to DAPI-positive cells. And each group was compared with the young group, respectively.

### Cell culture

The foreskin samples used in this study were donated by healthy adults aged 20–30 years who underwent surgical circumcision, and all donors were of the Asian race. Fresh foreskins were obtained with the donors’ consent and the approval of the Ethics Committee of the Fourth Medical Center of Chinese PLA General Hospital, Beijing, China. The foreskin tissue was surgically removed using aseptic techniques. The collected samples were kept in sterile PBS and transported at 2–8 °C to the laboratory for processing. To obtain the EpiSCs, subcutaneous fat was removed from skin samples with a scalpel, and skins were placed dermis side down in 2.4 U/mL Dispase II for 1 h. Skin was scraped to separate the epidermis from the dermis. Single-cell suspensions were obtained by trypsin digestion at 37 °C. Cells were then filtered through 70 mm, followed by 40 mm strainers, and plated on collagen IV-coated 6-well tissue culture dishes to establish primary cell lines as described [10]. Independent clones were cultured and passaged in Epilife culture medium (MEPI500CA, Gibco) with Human Keratinocyte Growth Supplement (HKGS) (S0015, Gibco). The 2nd and 3rd passages were used in the experiments. For experiments, cells were cultured in Epilife culture medium with or without Erlotinib (2 μM, HY-50896, MCE)), Tideglusib (200 nM, HY-14872, MCE) or EGF (20 ng/mL, P00033, Solarbio).

### Cell migration assays

A scratch-wound healing assay was performed essentially as described [11]. In brief, EpiSCs were plated on 6-well tissue culture dishes. After cells reached confluency, wounds were created by manual scraping of the cell monolayer with a pipette tip. The dishes were then washed with PBS, replenished with Epilife culture medium with different drugs, and photographed with a phase-contrast microscope. Afterward, dishes were placed in the tissue-culture incubator, and the matched wound regions were photographed 12, 24, and 36 h after wounding. The experiment was repeated three times independently. ImageJ software was used to measure the average width of the scratches. The migration rate of the scratch was calculated as follows: migration rate (%) = *(W*_*0*_* − W*_*t*_*)/W*_*0*_ × 100%, where *W*_*0*_ is the original width and *W*_*t*_ is the remaining width at the measured time point. The migration rate of each group was compared with the control group, respectively, at each time point.

### Sulforhodamine B assay

The effect of Erlotinib or Tideglusib on cell proliferation was investigated using the sulforhodamine B (SRB) assay (ab235935, Abcam). In brief, 10^4^ cells were seeded into sterile 96-well plates containing 100 μL of Epilife culture medium with HKGS and incubated 24 h for cell adherence, with 6 wells in each group. The wells were then washed with PBS and replenished with Epilife culture medium with different drugs for 12, 24, and 36 h. At indicated time points after treatment, cells were fixed with pre-cooled trichloroacetic acid and incubated at 4 °C for 1 h. The plates were then washed 3 times with deionized water and stained with SRB dye (0.4% dissolved in 1% acetic acid) for 25 min at room temperature. Wells were then washed 3 times with 1% acetic acid to remove any unbound dye and dried in the air at RT. 100 μL Tris (10 mM) base was added to each well to solubilize bound dye and gently mixed to obtain a homogenous solution. Finally, absorbance was measured at 510 nm wavelength by a microplate reader (Synergy2, BioTek) per the manufacturer’s instructions. The optical density (OD) value of each group was compared with the control group, respectively, at each time point.

### CCK-8 assay

The cytotoxicity of Tideglusib on EpiSCs was detected by CCK-8 assay. Briefly, the 2nd and 3rd passages of EpiSCs were seeded into a 96-well plate, with 6 wells in each group. After incubated with Epilife culture medium with different concentration of Tideglusib for 24 h, cells were replenished with 10 μL CCK-8 reagent (HY-K0301, MCE) and then incubated at 37 °C for 2 h before a microplate reader (Synergy2, BioTek) was employed to measure the absorbance at 450 nm wavelength. The OD value of each group was compared with the control group, respectively.

### Flow cytometry

To detect cell apoptosis and cell cycle, preparation of EpiSCs and staining protocols were done with Annexin V-FITC Apoptosis Detection Kit I (556547, BD) and PI/RNase Staining Buffer (550825, BD) per manufacturer’s instructions. The experiment was repeated 3 times independently. The apoptosis rate of each group was calculated as the sum of the ratios of early apoptosis and late apoptosis and compared with the control group, respectively.

### Quantification and statistical analysis

The analysis was performed in a blinded fashion. The statistical analysis was performed by two-tailed unpaired Student’s *t* test, one-way analysis of variance (ANOVA) followed by the multiple comparisons Bonferroni test and presented as the mean ± SD using the Prism software (GraphPad). For all statistical tests, the 0.05 level of confidence was accepted as a significant difference.

## Results

### Transcriptome changes in skin aging

To explore the potential mechanism underlying the senescence of skin, we analyzed the RNA-Seq data of young and aged skin of mice obtained from GEO Series GSE35322. Heatmap and volcano map were generated to show the general characteristics of mRNA profiles changing with age (Fig. 1a, b). With differentially expressed genes screened, the up- or downregulated genes were, respectively, subjected to KEGG analysis to identify age-related changes in signaling pathways (Fig. 1c, d). Among upregulated genes and pathways, only ubiquitin-mediated proteolysis, cAMP signaling pathways, and adherent junction showed age-related changes with *P* < 0.05 (Fig. 1c). On the contrary, more signaling pathways manifested age-related decline with *P* < 0.05 (Fig. 1d). Among them, PI3K/Akt signaling pathway was closely related to cell proliferation and apoptosis in response to extracellular signals, which played an important role in the development and maintenance of epidermis and EpiSCs.

### Age-related changes in the Akt/mTOR pathway

To further verify the change of PI3K/Akt signaling pathway with age, epidermis from rats of different age was collected and subjected to Western Blot. Compared with 2M group, the phosphorylation of Akt and its downstream mTOR in the epidermis of 17M group was slightly reduced (68.7 ± 15.8%, 78.9 ± 13.9%) but not statistically different (*P* > 0.05, Fig. 1e). The activity of the PI3K/Akt pathway decreased obviously in 23M epidermis, indicated by reduced ratio of p-Akt (32.1 ± 13.8%) and p-mTOR (38.9 ± 11.8%) compared to 2M group (*P* < 0.01, Fig. 1e). As the downstream of PI3K/Akt, Cyclin D1 plays a key role in regulating the cell cycle and cell proliferation. As shown in Fig. 1e, the expression of Cyclin D1 protein in epidermis decreased with age from 64.2 ± 17.5% of 17M to 36.8 ± 14.4% of 23 M (*P* < 0.05, Fig. 1e). Apoptosis is also regulated by the PI3K/Akt pathway, and the increase in the ratio of Bax to Bcl2 indicates the enhancement of apoptosis. We found that the ratio of Bax to Bcl2 continued to increase with age from 2.15 ± 0.38 times of 17M to 3.23 ± 0.41 times of 23M (*P* < 0.01, Fig. 1e). As quantified in immunofluorescence of the mice epidermis, significantly fewer Cyclin D1, more TUNEL-labeled cells were seen in old versus young epidermis (*P* < 0.01), indicating a decrease in proliferation and an increase in apoptosis (Fig. 1f).

### Declines in EGFR expression restricted the activation of PI3K/Akt pathway and wound healing in aged epidermis

By activating receptor tyrosine kinases causing autophosphorylation, a variety of growth factors and signaling complexes can initiate activation of the PI3K/Akt signaling pathway, including epidermal growth factor (EGF), fibroblast growth factor (FGF), vascular endothelial growth factor (VEGF), hepatocyte growth factor (HGF), angiopoietin I (Ang1) and insulin. As for EpiSCs, the PI3K/Akt pathway can be activated by the ligand EGF via its membrane receptor EGFR, which in turn triggers the activation of mTOR. EGF and EGFR play an important role in the homeostasis maintenance of epidermis and EpiSCs [12, 13]. To probe the underlying mechanisms of the decreased activation of PI3K/Akt, the expression of the upstream protein EGFR was detected. Quantified in Western Blot, EGFR expression in 17M (50.7 ± 12.5%) and 23 M (32.1 ± 13.8%) rats was significantly lower than that in 2M rats (*P* < 0.05, Fig. 2a). As shown in immunofluorescence, the expression of EGFR was visible in the epidermis of young mice, but thereafter, skins of middle (36.7 ± 4.3%) and old (27.6 ± 5.5%) mice exhibited little or no signs of EGFR (Fig. 2b). Consistent with the age-related expression trend of EGFR in rats and mice, the old human had lower fluorescence intensity of EGFR compared to the young human (25.8 ± 9.3%, *P* < 0.01, Fig. 2c).

To verify the relationship between EGFR expression and the activation of PI3K/Akt pathway in EpiSCs and wound healing, in vitro and in vivo experiments were performed with Erlotinib, an EGFR blocker. First, EpiSCs were extracted for experiments to exclude the influence of other cells in the epidermis. Compared to the control group, the PI3K/Akt pathway was affected by the blocked EGFR and manifested as a decrease in phosphorylation of Akt (30.4 ± 10.6%) and mTOR (39.8 ± 12.8%) (*P* < 0.01, Fig. 2d). Cyclin D1 expression was also impaired by the EGFR blocker (20.9 ± 8.7%, *P* < 0.01, Fig. 2d) and the ratio of BAX to Bcl2 increased (3.18 ± 0.33 times, *P* < 0.01, Fig. 2d). In the cell migration assays, EpiSCs incubated with 2 μM Erlotinib showed a significant delay in migration and scratch closure on 24 h and 36 h post-injury (*P* < 0.01, Fig. 2e). The SRB assay also reflected the decrease in cell proliferation with EGFR blocked 24 h and 36 h (*P* < 0.05, Fig. 2f). The cell cycle and apoptosis were assayed by flow cytometry after 24-h incubation of 2 μM Erlotinib (Fig. 2g, h). Corresponding reduction in proliferation manifested as a decreased ratio of (1-G1) / G1 (Fig. 2g) and the elevated apoptosis rate from 8.92 ± 0.32 to 11.88 ± 1.71% revealed the weakened ability of EGFR-blocked EpiSCs to survive (*P* < 0.05, Fig. 2h).

**Fig. 3:**
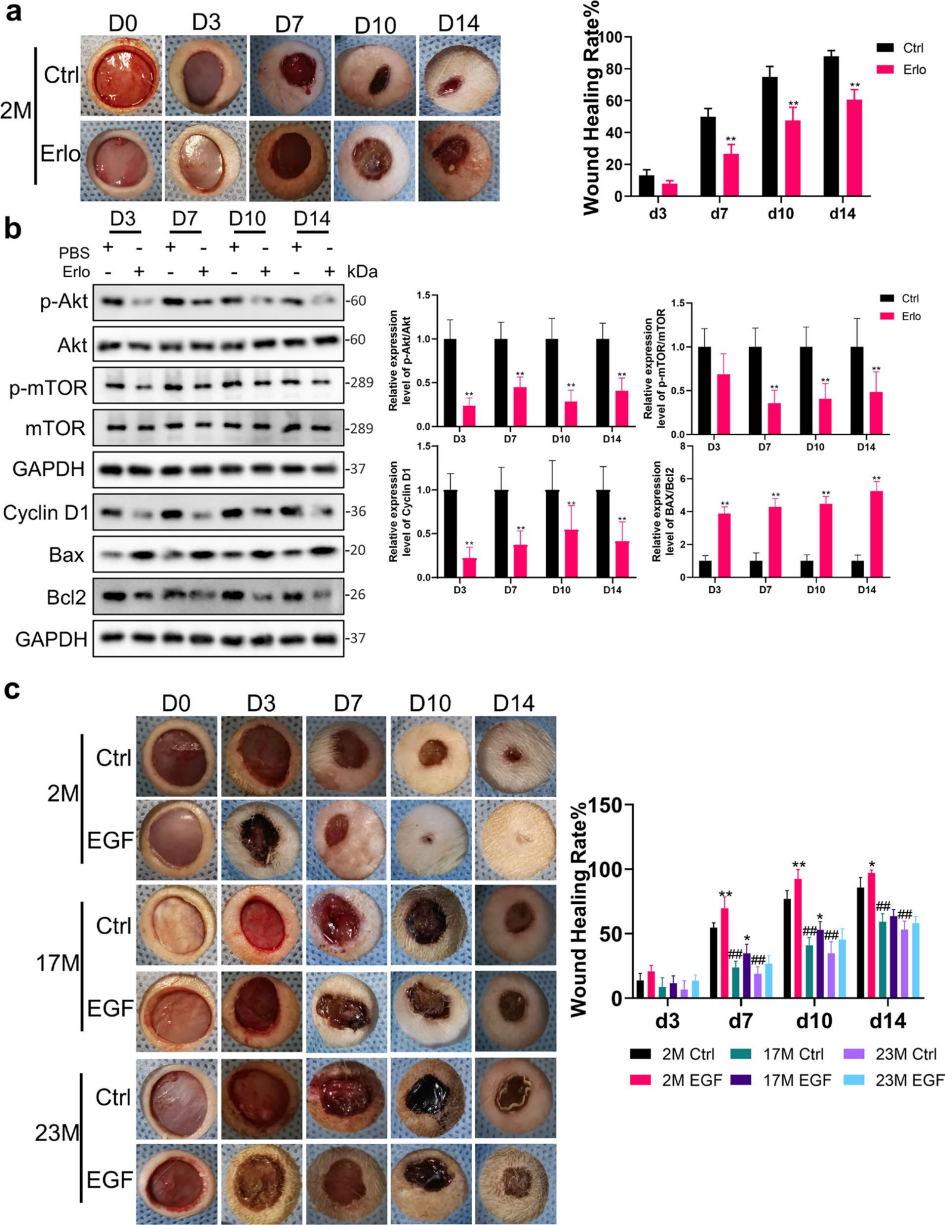
Declines in EGFR expression restricted wound healing and EGF efficacy in aged epidermis. **a** Gross view of wounds with different treatments post-wounding. *N* = 6. *P < 0.05, **P < 0.01 vs. the control group. **b** 1 mm epidermis samples from the wound margin of 2 M SD rats were analyzed by Western Blot. *N* = 6. *P < 0.05, **P < 0.01 vs. the control group. **c** We generated 10 mm full-thickness cutaneous wounds on the backs of 2 M, 17 M and 23 M rats, and animals of each age were randomly divided into control group and EGF-treated group that received a total 200 μL drug (PBS, 10 μg/mL EGF) sprayed evenly on the wound everyday post wounding. *N* = 6. *P < 0.05 and **P < 0.01 were used to indicate the difference between the control group and the EGF-treated group within the rats of each age at time points. #*P* < 0.05 and ##*P* < 0.01 were used to indicate the difference between the control group of 2 M, 17 M and 23 M rats at time-points

Next, we blocked the function of EGFR in 2M rats to verify the relationship between EGFR, PI3K/Akt pathway, and wound healing process. As shown in the images from representative experiments, compared with the control group, the wound healing rate of Erlotinib-treated group decreased significantly at d7 from 49.84 ± 5.32 to 26.72 ± 5.71%, d10 from 74.87 ± 6.61 to 47.58 ± 8.30% and d14 from 87.98 ± 3.48 to 60.52 ± 6.47% (*P* < 0.01, Fig. 3a). Consistent with our punch wound studies, we also observed a notable decrease in the phosphorylation of Akt (*P* < 0.01) and mTOR in Erlotinib-treated group during wound healing, accompanied by an impaired expression of Cyclin D1 (*P* < 0.01) and increased apoptosis ratio of BAX to Bcl2 (*P* < 0.01, Fig. 3b). The delayed wound closure in Erlotinib-treated animals corresponded well to the decreased activation of PI3K/Akt signaling pathway.

### Declines in EGFR expression restricted the efficacy of EGF in aged skin

By binding to the membrane receptor EGFR, EGF is widely used in clinical to accelerate wound healing. Considering the reduced expression of EGFR in aged skin, we conducted experiments to explore the age-related changes in the efficacy of EGF on wound healing process. As shown in the Fig. 3c 2M rats consistently closed their wounds faster than their aged counterparts, and aged skin was slow to re-epithelialize wounds following injury (Fig. 3c). From the perspective of EGF efficacy, EGF had an obvious effect on 2 M rats whose wound healing rate was accelerated from d7 (69.81 ± 8.71 to 54.63 ± 3.85%) to d14 (97.17 ± 2.23 to 85.78 ± 7.72%) (*P* < 0.05, Fig. 3c). Nevertheless, the improvement in wound healing rate of 17 M rats by EGF was statistically significant only at d7 (34.63 ± 7.13 to 23.98 ± 4.70%) and d10 (52.89 ± 6.41 to 41.12 ± 5.94%) (*P* < 0.05, Fig. 3c). The wound healing rate was not statistically different between the control group and the EGF treatment group of old rats at any time point (*P* > 0.05, Fig. 3c).

### Tideglusib promoted PI3K/Akt phosphorylation in EpiSCs

**Fig. 4:**
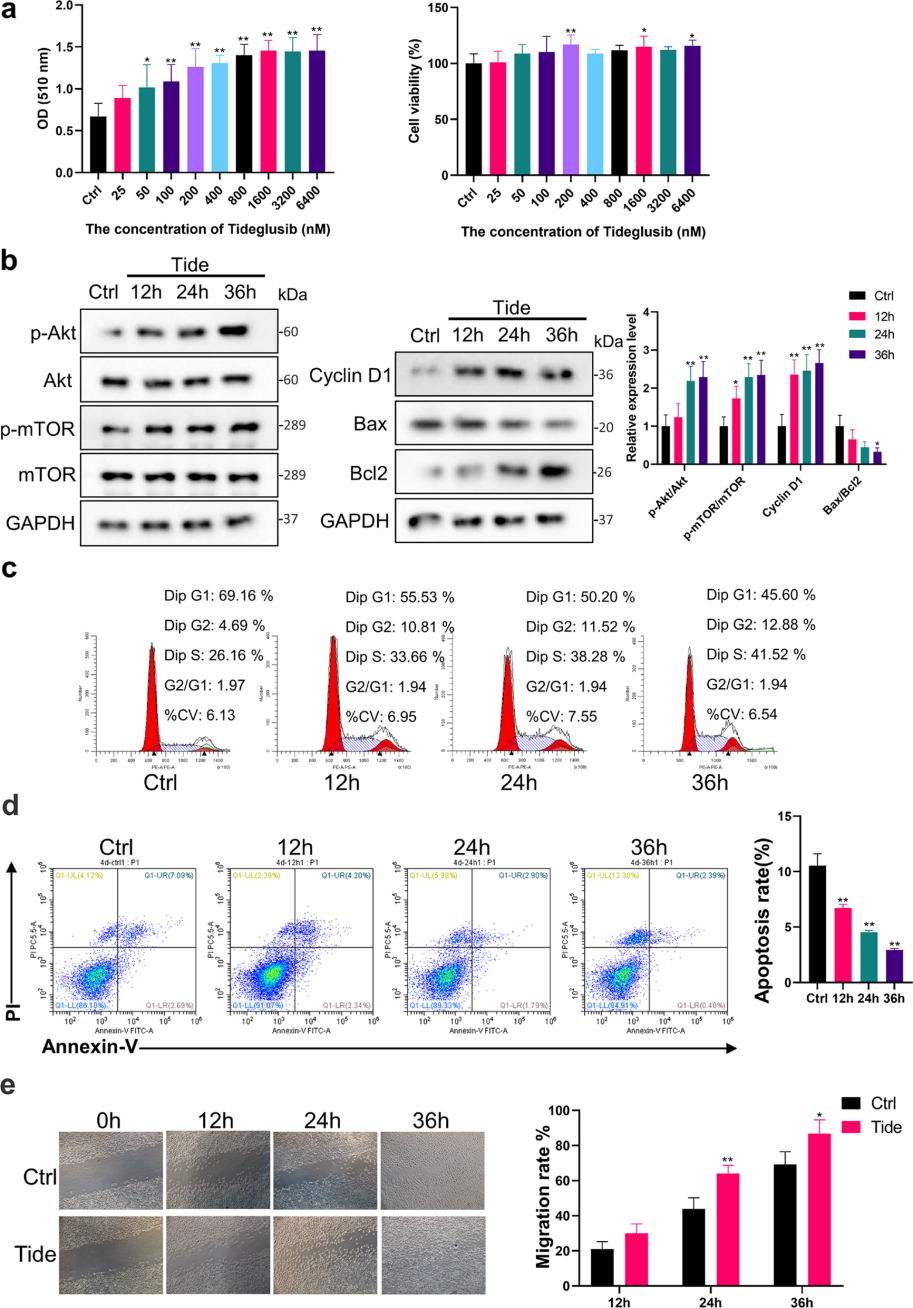
Tideglusib promoted PI3K/Akt phosphorylation in EpiSCs. **a** The proliferation effect and toxicity of different concentrations of Tideglusib on EpiSCs were compared by the SRB assay and CCK-8 experiment. *P < 0.05, **P < 0.01 vs. the control group. *N* = 6. **b** The phosphorylation of Akt and mTOR, the proliferation related protein Cyclin D1, the apoptosis related protein Bax and Bcl2 were assessed by Western Blot. *N* = 3 for each group. *P < 0.05, **P < 0.01 vs. the control group. **c** and **d** Flow cytometry was further used to quantify cell cycle distribution and apoptosis in cells treated with PBS or 200 nM Tideglusib. *P < 0.05, **P < 0.01 versus the control group. *N* = 3 for each group. **e** Confluent monolayers of EpiSCs were randomly divided into two groups that received PBS or 200 nM Tideglusib and subjected to in vitro scratch-wound assays. Quantifications of wound closure are shown in the graph right. *N* = 3 for each group. *P < 0.05, **P < 0.01 versus the control group at time-point

Our collective findings pointed out the functional importance of PI3K/Akt pathway in wound repair. Trapped by reduced EGFR expression, a decline in the ability of EGF to activate the pathway is at the crux of the age-related wound repair defects, and it should be possible to enhance wound healing in aged skin through an PI3K/Akt signaling pathway agonists (summarized in Fig. 7c). It has been reported that the small molecule drug Tideglusib can activate the PI3K/Akt signaling pathway [14]. Since the drug does not require a membrane receptor to work, we conducted experiments to see if it could promote PI3K/Akt phosphorylation in EpiSCs. Tideglusib could promote cell proliferation in the concentration range from 50 to 6400 nM (*P* < 0.05) and even 6400 nM Tideglusib was not cytotoxic to EpiSCs (Fig. 4a). After 36-h incubation, 200 nM Tideglusib elevated the phosphorylation of Akt (2.29 ± 0.41 times) and mTOR (2.35 ± 0.39 times), indicating the activation of the PI3K/Akt pathway (*P* < 0.01, Fig. 4b). Expression of Cyclin D1 was also enhanced (2.66 ± 0.35 times) and the ratio of BAX to Bcl2 reduced (0.33 ± 0.10 times) (*P* < 0.05, Fig. 4b). The flow cytometry data revealed the corresponding enhanced proportion of cells in mitosis and inhibited apoptosis (Fig. 4c, d). Simultaneously, the migration ability of EpiSCs incubated with Tideglusib was enhanced from 24 to 36 h (Fig. 4e).

### Tideglusib improved wound repair in aged skin

**Fig. 5:**
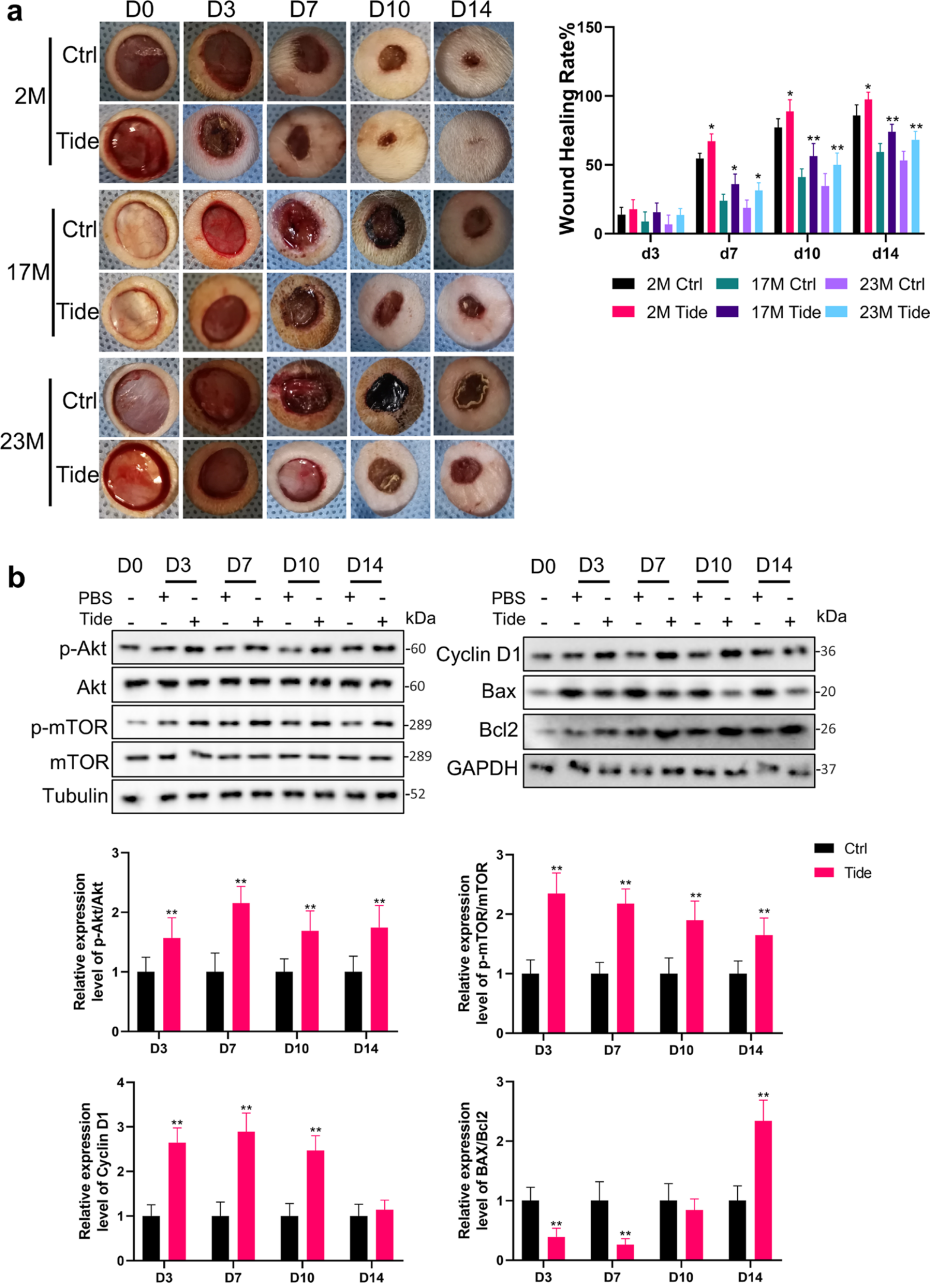
Tideglusib improves wound repair in aged skin. **a** Gross view of wounds with different treatments post-wounding. *N* = 6. *P < 0.05 and **P < 0.01 were used to indicate the difference between the control group and the Tideglusib-treated group within the rats of each age at time-points. **b** 1 mm epidermis samples from the wound margin of 23 M SD rats were analyzed by Western Blot. The sample size of each treatment group at each time point is 6. *P < 0.05, **P < 0.01 vs. the control group at time-point

Punch wounds were administered on rats of different ages to test the effect of Tideglusib on wound healing. At d3 post-injury, the difference in wound healing rate between Tideglusib treatment group and the control group was not statistically significant (*P* > 0.05, Fig. 5a), probably because the wound was still in the inflammatory reaction stage. At d7 post-injury, the wound healing rate of 2M rats in Tideglusib group was 67.12 ± 2.15% higher than the 54.63 ± 1.57% of the control group (*P* < 0.05). Meanwhile, the wound healing rate of 17M and 23M rats increased from 23.98 ± 1.92 to 35.93 ± 2.99%, and 18.76 ± 2.33 to 31.41 ± 2.27%, respectively (*P* < 0.05, Fig. 5a). This trend continued from d10 to d14 post-injury, manifested by a faster wound healing rate in the Tideglusib treatment group in 2M, 17M, and 23M rats (*P* < 0.05, Fig. 5a). Corroborating the wound healing rate, Tideglusib elevated the phosphorylation of Akt and mTOR in 23M rats after wounding from d3 to d14 (*P* < 0.01, Fig. 5b). Synchronously, the expression of Cyclin D1 elevated from d3 to d10 (*P* < 0.01, Fig. 5b), and the ratio of BAX to Bcl2 decreased at d3, d7 and d14 (*P* < 0.01, Fig. 5b). These data reinforced the efficacy of Tideglusib in the wound healing of rats, especially the old independent of EGFR.

### The combined use of tideglusib and EGF accelerates wound healing in aged skin

As a clinically used drug, EGF showed a promotion effect on wound healing in young rats, but had little effect on wound healing in aged rats, which subjected to reduced EGFR expression in aging skin. EGF and Tideglusib activate the PI3K/Akt pathway, respectively, via cell membrane surface receptors or directly into cells. Thus, we took insights into exploring whether the combined use of Tideglusib and EGF could better promote the proliferation and migration of EpiSCs and wound healing in aged rats.

**Fig. 6:**
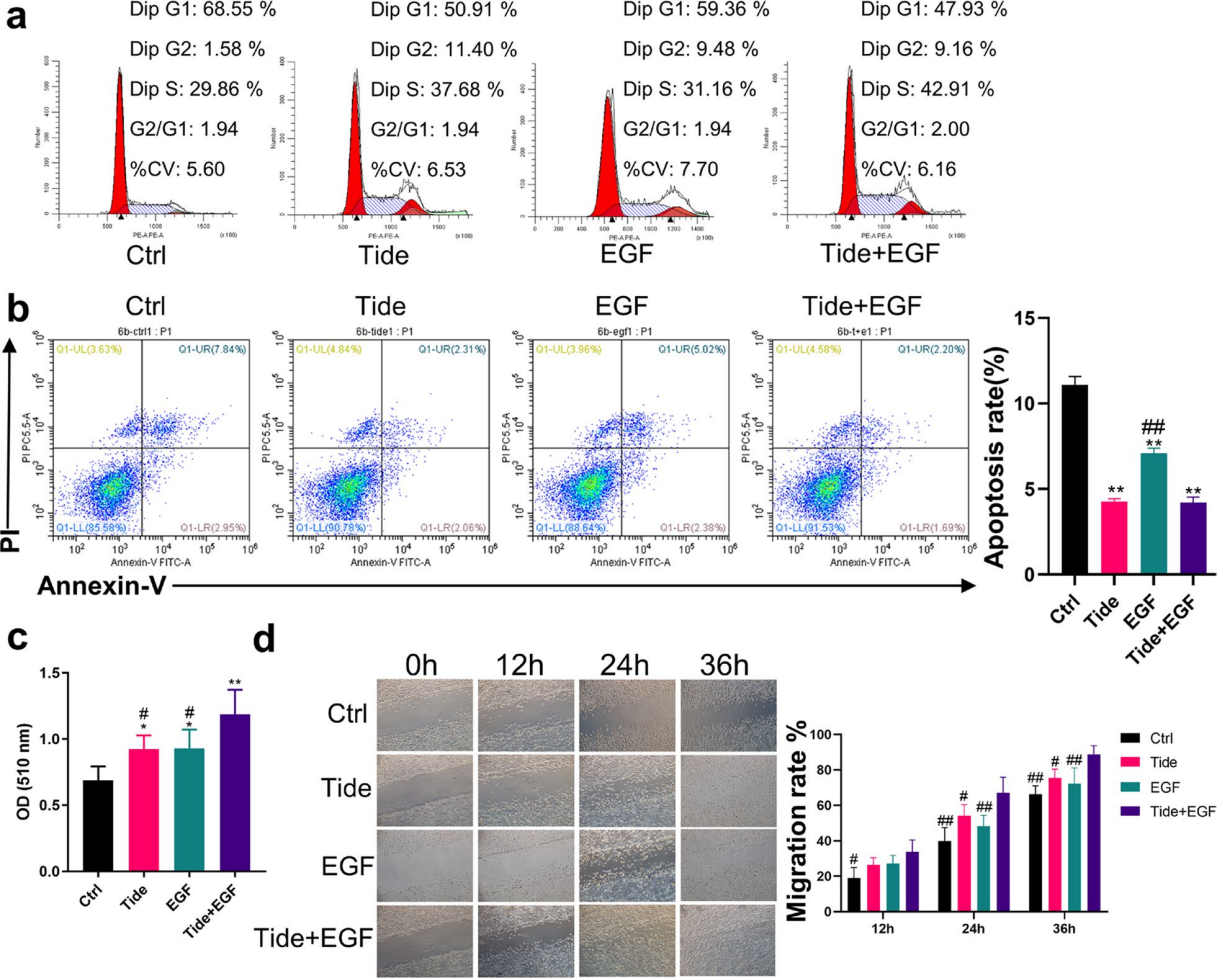
The combined use of Tideglusib and EGF could accelerate proliferation and migration of EpiSCs. EpiSCs were randomly divided into four groups that incubated with PBS, 200 nM Tideglusib, 20 ng/mL EGF, or combined use of Tideglusib and EGF dissolved in Epilife medium for 24 h. **a** and **b** Flow cytometry was used to quantify cell cycle distribution and apoptosis in cells treated with different drugs for 24 h. *P < 0.05 and **P < 0.01 vs. the control group. #*P* < 0.05, ##*P* < 0.01 versus the combination therapy group. *N* = 3. **c** SRB assay was performed to detect the proliferation effect of drugs on EpiSCs. *P < 0.05 and **P < 0.01 versus the control group. # *P* < 0.05, ## *P* < 0.01 versus the combination therapy group. *N* = 6. **d** Confluent monolayers of EpiSCs were randomly divided into four groups that received PBS, 200 nM Tideglusib, 20 ng/mL EGF, or combined use of Tideglusib and EGF dissolved in Epilife medium and subjected to in vitro scratch-wound assays. *N* = 3. #*P* < 0.05, ##*P* < 0.01 versus the combination therapy group at time-point

As for EpiSCs, compared with EGF or Tideglusib group at 24 h, the combined treatment group showed more cells in the DNA synthesis phase and lower apoptosis rate (*P* < 0.05, Fig. 6a, b). The SRB assay also showed the stronger proliferative capacity of the combined treatment group at 24 h (*P* < 0.05, Fig. 6c). Consistent with these data, the scratch healing ability of EpiSCs in the combined treatment group was enhanced compared with EGF or Tideglusib group at 24 h and 36 h after injury (*P* < 0.05, Fig. 6d).

**Fig. 7:**
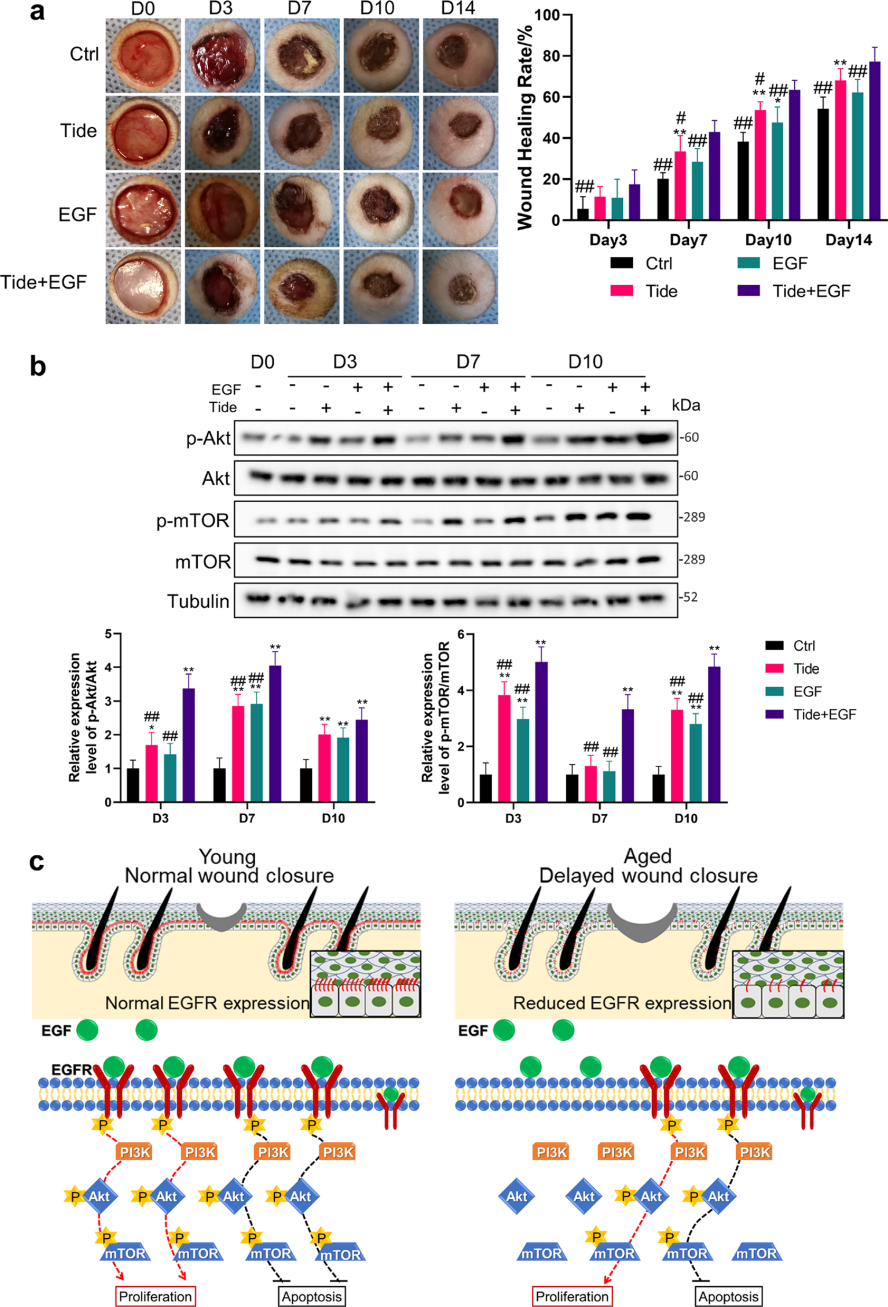
The combined use of Tideglusib and EGF accelerates wound healing in aged skin. We generated 10 mm full-thickness cutaneous wounds on the backs of 23 M rats, and animals were randomly divided into four groups that received a total 200 μL drug (PBS, 20 μM Tideglusib, 10 μg/mL EGF, or combined use of Tideglusib and EGF) sprayed evenly on the wound everyday post wounding. The blue fenestrated sheet has a diameter of 12 mm and is used as a reference. *N* = 6. *P < 0.05 and **P < 0.01 were used to indicate the difference between the control group and the other groups. #*P* < 0.05, ##*P* < 0.01 were used to indicate the difference between the combination therapy group and the other groups. **a** Gross view of wounds with different treatments post-wounding. **b** 1 mm epidermis samples from the wound margin of 23 M SD rats were analyzed by Western Blot. *N* = 6. **c** Schematics depict the re-epithelialization process in young and aged animals. Owing to the decreased expression of EGFR, self-secreted or externally administered EGF could not effectively bind to its membrane receptor EGFR and thus cannot effectively induce sufficient phosphorylation of EGFR. Therefore, aged skin exhibits reduced PI3K/Akt pathway activity, lower proliferation capacity and higher levels of apoptosis. While wounds of young animals heal, the process is delayed in aged skin and could not be effectively restored by EGF

Finally, we examined wound repair in 23M rats to verify the efficacy of combined treatment. At d3 post-injury, compared with the control group, Tideglusib or EGF treatment alone did not show a statistical difference while the combined treatment group had an obvious improvement from 5.67 ± 5.83 to 17.43 ± 7.03% (*P* < 0.01, Fig. 7a). From d7 to d10, the efficacy of the combined treatment group was always better than the other 3 groups (*P* < 0.05, Fig. 7a). At d14 post-injury, while there was no statistical difference compared with the Tideglusib group (68.06 ± 2.34%), the wound healing rate of the combined treatment group (77.20 ± 2.88%) was still better than that of the control group (54.29 ± 2.32%) and the EGF group (62.24 ± 2.57%) (*P* < 0.01, Fig. 7a). Verified in Western Blot, compared with the EGF group and the Tideglusib group, the combined treatment group had more phosphorylation of Akt at d3 and d7 (*P* < 0.01, Fig. 7b), and mTOR at d3, d7, and d10 (*P* < 0.01, Fig. 7b) post injury.

## Discussion

Wound healing requires the interaction of various signaling pathways, which must be coordinated in a spatiotemporal manner to achieve proper re-epithelialization [7]. Poor wound healing was first observed in older soldiers in World War I [15], and more rigorous experimental evidence in rats and other animals has also revealed delayed wound healing in aged tissues [16–20]. Delayed wound healing in aged adults contributes to various health complications and decreased lifespan, which needs to be solved urgently [21].

By analyzing the transcriptomic data of mice skin of different ages from the GEO database, we focused on the epidermis and EpiSCs-related signaling pathways in differentially expressed genes. As a classical pathway regulating the proliferation and apoptosis of EpiSCs, PI3K/Akt signaling pathway can accelerate wound healing via regulating its downstream effector, like mTOR, IKK, MDM2, etc. KEGG analysis showed that PI3K/Akt signaling pathway manifested significant age-related differences. Verified by Western Blot and immunofluorescence, we found that the activation of PI3K/Akt signaling pathway decreased with age, and the expression of its downstream Cyclin D1 decreased with apoptosis enhanced. PI3K/Akt signaling pathway in EpiSCs was mainly regulated by EGFR-initiated phosphorylation cascade. Furthermore, we found that the expression of EGFR in the skin of mouse, rat, and human all decreased with age. The expression level of EGFR affects cell metabolism. Mice with increased EGFR expression demonstrate enhanced PI3K/Akt signaling, faster reproduction of EpiSCs, and increased epidermal thickness [22]. Keratinocytes with blocked EGFR showed an increase in senescent cells [23]. Mice with knockout of EGFR in the skin displayed increased inflammation, degeneration of hair follicles, impaired skin barrier function, and slower cell proliferation [24, 25]. Through in vitro and in vivo EGFR blocking experiments, we confirmed that decreased EGFR expression in aged skin leads to decreased activation of PI3K/Akt signaling pathway, which in turn leads to the disturbance of downstream cell proliferation and apoptosis of EpiSCs, and thus affects the wound healing rate of animals.

Discovered by American scientist Dr. Stanley Cohen in the 1960s, EGF is nowadays one of the most commonly used drugs to promote wound repair in clinical practice [26]. EGF has shown its superiority in promoting proliferation and angiogenesis of vascular endothelial cells, collagen synthesis of fibroblasts, and migration and differentiation of EpiSCs [27–30]. However, studies have shown that EGF has a poor effect on senescent fibroblasts [31–34]. Age-related changes in the efficacy of EGF on EpiSCs and wound healing have not been well reported before. In this study, we found that EGF cannot effectively improve the wound healing rate of aged rats, and its efficacy showed age-related decline. The molecular underpinnings of the age-related efficacy changes in wound healing may be attributed to the decreased expression of EGFR, which led to the restricted activity of PI3K/Akt pathway in EpiSCs.. Considering the changes of EGFR with skin aging and the importance of PI3K/Akt signaling pathway to wound healing, looking for drugs that can activate the pathway may promote wound healing in aged skin.

Small molecule drugs feature excellent controllability in time and space of administration. By far, some small molecule drugs have been extensive used in clinical practice, such as Revlimid for multiple myeloma, Imbruvica for lymphoma, and Palbociclib for breast cancer. In terms of Tideglusib, 5 phase II clinical trials have been completed in the UK, USA, Canada, France, and Germany, and 1 phase III clinical trial was in progress. Studies demonstrated that Tideglusib was generally safe and well-tolerated with no early discontinuations due to adverse events or dose adjustments [35]. Previous studies have shown that Tideglusib can not only be used to treat Alzheimer’s disease and myotonic muscular dystrophy, but also promote dentine regeneration [36, 37]. Study has also revealed Tideglusib’s ability in activating PI3K/Akt signaling pathway in myotubes, hypoxic-ischemic brain injury, etc. [14, 38, 39]. In this study, we found that Tideglusib could promote the activation of PI3K/Akt signaling pathway in EpiSCs, thus accelerating wound healing not only in young rats but also in aged rats.

The expression of EGFR is modulated by various factors, such as ligand-induced endocytosis, phosphorylation, and ubiquitination, and the balance of positive and negative feedback maintains the normal expression level of EGFR [40, 41]. It has been reported that EGF can trigger the rapid activation of EGFR, but what EGF triggers is not sustainable, but temporary EGFR signaling owing to the negative feedback of EGF/EGFR axis [42]. In the process of wound healing, the proliferation and migration of cells at the wound site require sustained signaling, and temporary EGFR signaling may impose restrictions on the speed of wound healing. In the aged skin, the decreased expression of EGFR and the negative feedback of EGF/EGFR may both disrupt wound healing. As Tideglusib could activate PI3K/Akt signaling pathway independent of EGFR, we explored whether the combined use of Tideglusib and EGF could promote wound healing more effectively. The results showed that EpiSCs displayed stronger proliferation and lower apoptosis after combined treatment with Tideglusib and EGF. In vivo experiments confirmed that the PI3K/Akt/mTOR activity of the combined treatment group was higher than those of the EGF group, and the wound healing rate was faster. This indicates that the combined use of Tideglusib and EGF may help accelerate re-epithelialization in the process of wound healing.

In summary, through the analysis of skin transcriptome sequencing data of different ages, we found changes in the PI3K/Akt pathway and its upstream EGFR during skin aging. Through wound healing experiments in rats of different ages, we confirmed the age-related decrease in the efficacy of EGF, suggesting that EGF may not be a foolproof option for clinicians when treating wounds in elderly patients. Furthermore, we proposed and demonstrated that the membrane receptor-independent activator of the PI3K/Akt pathway, Tideglusib, could be used to promote wound healing in aged rats. Meanwhile, we further proved that the combined use of EGF and Tideglusib could better promote wound healing in aged rats, which provided a new idea for clinical treatment. Clinicians can consider comprehensive application of multiple drugs when treating elderly patients with wounds for better curative effect.

In addition, this study has the following shortcomings which need to be further improved: (1) The sequencing data used in this study were derived from mouse skin, which could not fully reflect the aging of human skin. Further transcriptome sequencing or single-cell sequencing of human skin of different ages is urgently needed to better explain the mechanism of delayed wound healing in aged skin. (2) Based on sequencing data, this study only explored the age-related changes of PI3K/Akt pathway and EGFR. The effects of other age-related pathways on wound healing require further study, such as IL-17 pathway and ubiquitination mediated proteolysis. (3) The reasons why the expression of EGFR decreased in aged skin has not been traced and reported. It is urgent to further explore the age-related changes of EGFR-related transcriptional regulators and ubiquitination-related proteins. (4) The specimens used in this study were all male while males and females may be inconsistent in the skin aging and the mechanisms of delayed wound healing, which need further detection.

## Conclusions

Aging affects the skin, especially the epidermis via the disturbed PI3K/Akt signaling pathway, owing to the decreased expression of EGFR. Our finding demonstrates that Tideglusib, a small molecule drug that has entered clinical trials, can activate PI3K/Akt signaling pathway. The combined use of Tideglusib and EGF can be used to promote wound healing in aged adults. Our study demonstrates a potential therapeutic for wound healing in the old which needs further research.

## Data Availability

Further information and requests for reagents may be directed to and will be fulfilled by the Lead Contact: Chuan’an Shen (shenchuanan@126.com).
